# Prognostic value of loss of heterozygosity and sub-cellular localization of SMAD4 varies with tumor stage in colorectal cancer

**DOI:** 10.18632/oncotarget.15560

**Published:** 2017-02-21

**Authors:** Xu Jia, Chandrakumar Shanmugam, Ravi K. Paluri, Nirag C. Jhala, Michael P. Behring, Venkat R. Katkoori, Shajan P. Sugandha, Sejong Bae, Temesgen Samuel, Upender Manne

**Affiliations:** ^1^ Department of Pathology, University of Alabama at Birmingham, Birmingham, AL, USA; ^2^ Department of Medicine, University of Alabama at Birmingham, Birmingham, AL, USA; ^3^ Department of Epidemiology, University of Alabama at Birmingham, Birmingham, AL, USA; ^4^ College of Veterinary Medicine, Tuskegee University, Tuskegee, AL, USA; ^5^ Comprehensive Cancer Center, University of Alabama at Birmingham, Birmingham, AL, USA; ^6^ Current address: Department of Pathology, ESIC Medical College and Hospital, Sanathnagar, Hyderabad, Telangana, India; ^7^ Current address: Pathology & Laboratory Medicine, Temple University, Philadelphia, PA, USA; ^8^ Current address: Department of Surgery, Michigan State University, College of Human Medicine, Lansing, MI, USA

**Keywords:** colorectal cancer, tumor stage, 18q21 LOH, nuclear/cytoplasmic SMAD4, survival

## Abstract

**Background:**

Although loss of heterozygosity (LOH) at chromosome location *18q21* and decreased expression of SMAD4 in invasive colorectal cancers (CRCs) correlate with poor patient survival, the prognostic value of LOH at *18q21* and sub-cellular localization of SMAD4 have not been evaluated in relation to tumor stage.

**Methods:**

Genomic DNA samples from 209 formalin-fixed, paraffin-embedded sporadic CRC tissues and their matching controls were analyzed for *18q21* LOH, and corresponding tissue sections were evaluated by immunohistochemistry for expression of SMAD4 and assessed for its sub-cellular localization (nuclear vs. cytoplasmic). In addition, 53 frozen CRCs and their matching control tissues were analyzed for their mutational status and mRNA expression of SMAD4. The phenotypic expression pattern and LOH status were evaluated for correlation with patient survival by the use of Kaplan-Meier and Cox regression models.

**Results:**

LOH of *18q21* was detected in 61% of the informative cases. In 8% of the cases, missense point mutations were detected in *Smad4*. In CRCs, relative to controls, there was increased SMAD4 staining in the cytoplasm (74%) and decreased staining in the nuclei (37%). LOH of *18q21* and high cytoplasmic localization of SMAD4 were associated with shortened overall survival of Stage II patients, whereas low nuclear expression of SMAD4 was associated with worse survival, but only for patients with Stage III CRCs.

**Conclusions:**

LOH of *18q21* and high cytoplasmic localization of SMAD4 in Stage II CRCs and low nuclear SMAD4 in Stage III CRCs are predictors of shortened patient survival.

## INTRODUCTION

In the United States, colorectal cancer (CRC) is the third most common cancer in both women and men; in 2008, it caused about 50,000 deaths. Since approximately 50% of all CRC patients die of metastatic disease that is not apparent at surgery [1], efforts are underway to discover molecular determinants that identify patients who are at risk of developing recurrent CRC following surgical resection. These individuals could benefit from adjuvant chemotherapy.

The *Smad4* (mothers against decapentaplegic homolog 4) gene is located at chromosome locus *18q21.1* [2, 3]. At some stages of CRC, there is loss of heterozygosity (LOH) at this locus, and such loss is associated with a poor prognosis for lymph node-negative (stage II) CRC patients [2, 4–6]. However, the prognostic value of LOH of *Smad4*, particularly in lymph node-positive (stage III) patients, remains controversial [5, 7–9]. There is a higher prevalence of *Smad4* mutations in CRCs [10, 11], particularly in those with distant metastases (35%) than in locally advanced tumors (~10%) [11]. Moreover, animal studies show that *Smad4* inactivation is involved in the malignant transformation of gastrointestinal (GI) adenomas [12], and, during tumor progression, there are reductions in *Smad4* mRNA levels [13]. Similarly, CRC patients with tumors expressing low levels of SMAD4 mRNA or protein or immunophenotypic expression levels have worse survival outcomes than CRC patients with tumors expressing high levels of SMAD4 [14, 15]. Other investigations, however, have found *Smad4* mutations in only a small proportion of CRCs [16–19].

*Smad4*, a member of the *Smad* family of proteins, is an intracellular transducer that mediates the transforming growth factor (TGF-β)-*Smad*-dependent signaling pathway and translocation of the TGF-β complex into the nucleus. In the nucleus, these signals inhibit the growth of colon epithelial cells by regulating genes related to cell proliferation, differentiation, and apoptosis [3, 11, 20–23]. In CRCs (all stages), low immunophenotypic expression of *Smad4* correlates with poor patient survival [17, 24–28]. Some recent reports show a poor prognostic value for low phenotypic expression of *Smad4* but no association for *18q21* allelic imbalance in CRCs [14]. These results, however, are contradictory to those reported for most of studies of CRCs [5, 8, 29–31].

The present study evaluated the role of *Smad4* alterations (mutations, LOH expression, and sub-cellular localization) in CRC tissues from patients who did not receive adjuvant chemotherapy. The association between the abnormal variations in *Smad4* and overall patient survival and by tumor stage was evaluated. The results provide evidence that SMAD4 is a prognostic marker for some patients with CRC.

## RESULTS

**Figure 1:**
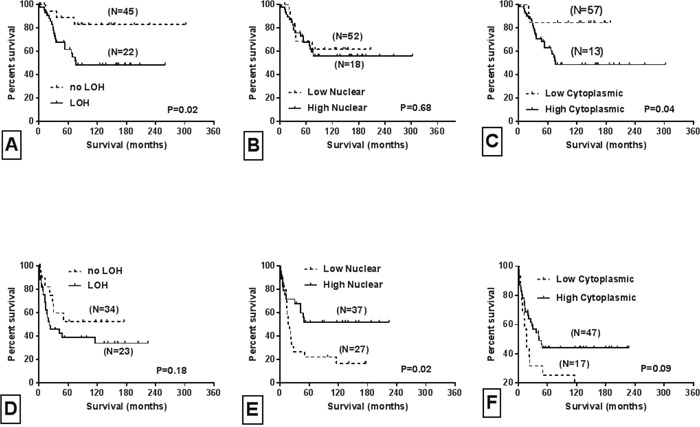
Univariate survival curves based on tumor stage, LOH and Smad4 sub-cellular localization Kaplan-Meier survival curves for stage II patients according to: **A**. LOH status, **B**. nuclear Smad4 expression, **C**. cytoplasmic Smad4 expression. Kaplan-Meier survival curves for stage III patients according to: **D**. LOH status, **E**. nuclear Smad4 expression, **F**. cytoplasmic Smad4 expression. Log rank P values are provided.

Fifty-five cases (55/209, 26%) and 77 cases (77/209, 37%) exhibited low SMAD4 immunohistochemical protein (IHC) expression in the cytoplasm and nucleus, respectively (Table 1). Kaplan-Meier survival analyses were conducted for nuclear and cytoplasmic protein expression for the entire study sample and by tumor stage. For the overall sample, there was a marginally statistically significant association between low nuclear SMAD4 IHC levels and poorer survival of these patients (log-rank, *P* = 0.07) (data not shown). There was, however, no association for patients with low SMAD4 IHC expression in cytoplasm (log-rank, *P* = 0.28) (data not shown). The 74 Stage II and 64 Stage III cases were then analyzed separately. For stage III patients, a low nuclear SMAD4 IHC level was significantly associated with shorter survival (log-rank, *P* = 0.02; Figure 1E). However, for Stage II patients, high cytoplasmic IHC expression of SMAD4 was associated with an increased risk of cancer-specific mortality (log-rank, *P* = 0.047; Figure 1C). These results suggest that, for stage III patients, low nuclear IHC expression of SMAD4 is a prognostic marker for shorter survival and recurrence. In contrast, a high cytoplasmic IHC level was associated with worse survival of stage II patients. There was no significant association between IHC expression of SMAD4 and age, sex, ethnicity, location, or histological grade (Table 1). However, there was a trend towards statistical significance for association between nuclear (p=0.08) and cytoplasmic (p=0.07) protein expression with tumor stage (Table 1).

**Table 1:** Characteristics of the study sample (n = 209) according to *18q21* LOH and protein expression

Characteristics of study sample	n (%)	*18q21* LOH		*P*	Nuclear Localization		*P*	Cytoplasmic Localization		*P*
		No n (%) 73 (38.9)	Yes n (%) 118 (61.1)		Low n(%) 77 (36.8)	High n (%) 132 (63.2)		Low n(%) 55 (26.3)	High n (%) 154 (73.7)	
**18q21 deletion**										0.74
**No LOH**	73 (38.9)	--	--		29 (37.7)	62 (47.0)	0.19	25 (45.5)	66 (42.9)	
**LOH**	118 (61.1)	--	--		48 (62.3)	70 (53.0)		30 (55.5)	88 (57.1)	
**Cytoplasmic staining**							**< 0.001**			
**Low**	55 (26.3)	--	--		45 (59.7)	9 (6.8)		--	--	
**High**	154 (73.7)	--	--		31 (40.3)	123 (93.2)		--	--	
**Age**				0.79			0.37			0.23
**< 65**	92 (44.0)	41 (45.1)	51 (43.2)		37 (48.0)	55 (41.7)		28 (50.9)	64 (41.6)	
**≥ 65**	117 (56.0)	50 (54.9)	67 (56.8)		40 (52.0)	77 (58.3)		27 (49.1)	90 (58.4)	
**Sex**				0.99			0.73			0.42
**Male**	108 (51.7)	47 (51.7)	61 (51.7)		41 (53.3)	67 (50.8)		31 (56.4)	77 (50.0)	
**Female**	101 (48.3)	44 (48.4)	57 (48.3)		36 (46.8)	65 (49.2)		24 (43.6)	77 (50.0)	
**Race**				0.84			0.41			0.85
**Caucasian**	127 (60.8)	56 (61.5)	71 (60.2)		44 (57.1)	83 (62.9)		34 (61.8)	93 (60.4)	
**African American**	82 (39.2)	35 (38.5)	47 (39.8)		33 (42.9)	49 (37.1)		21 (38.2)	61 (39.6)	
**Tumor Stage**				0.45			0.08			0.07
**Stage I**	34 (16.3)	16 (17.6)	18 (15.3)		14 (18.1)	20 (15.1)		8 (14.6)	26 (16.9)	
**Stage II**	70 (33.5)	25 (27.5)	45 (38.1)		18 (23.4)	52 (39.4)		13 (23.6)	57 (37.0)	
**Stage III**	64 (30.6)	30 (33.0)	34 (28.8)		25 (32.4)	39 (29.6)		17 (30.9)	47 (30.5)	
**Stage IV**	41 (19.6)	20 (22.0)	21 (17.8)		20 (26.0)	21 (15.9)		17 (30.9)	24 (15.6)	
**Tumor size**				0.91			0.75			0.68
**≤ 65**	160 (78.1)	69 (78.4)	91 (77.8)		61 (79.2)	99 (77.3)		44 (80.0)	116 (77.3)	
**> 65**	45 (21.9)	19 (21.6)	26 (22.2)		16 (20.8)	29 (22.7)		11 (20.0)	34 (22.7)	
**Tumor grade**				0.06			0.33			0.94
**Low**	149 (71.3)	71 (78.0)	78 (66.1)		58 (75.3)	91 (68.9)		39 (70.9)	110 (71.4)	
**High**	60 (28.7)	20 (22.0)	40 (33.9)		19 (24.7)	41 (31.1)		16 (29.1)	44 (28.6)	
**Tumor location**				0.05			0.75			0.92
**Rectum**	42 (20.1)	19 (20.9)	23 (19.5)		15 (19.5)	27 (20.4)		11 (20.0)	31 (20.1)	
**Distal colon**	64 (30.6)	21 (23.1)	43 (36.4)		26 (33.8)	38 (28.8)		18 (32.7)	46 (29.9)	
**Proximal colon**	103 (49.3)	51 (56.0)	52 (44.1)		36 (46.7)	67 (50.8)		26 (47.3)	77 (50.0)	

### LOH in chromosome site *18q21* at the *Smad4* locus

Genomic DNA was retrieved from 209 FFPE tissue blocks of CRCs and their corresponding normal tissues. Three microsatellite biomarkers (D18S474, D18S46 and D18S363) that surround the *Smad4* gene were used to analyze LOH status of *18q21*. In at least one of the three markers, LOH was detected in 118 of the 193 (61%) informative cases (Table 1). Forty (40/118, 34%) cases exhibited LOH at two or three microsatellite markers. Excluding those with microsatellite instability and the ‘not detectable’ cases, the highest percentage (68/118, 58%) of informative cases was found at the D18S363 locus. The other two biomarkers had values of 40% (49/121) for the D18S46 locus and 41% (48//118) for the D18S474 locus (data not shown). For all 193 CRC cases, there was no significant difference in survival between the cases with and without *Smad4* LOH (log-rank, *P* = 0.14) (data not shown). Survival, however, was significantly associated with *Smad4* LOH for 67 stage II patients (log-rank, *P* = 0.02; Figure 1A), but not for 57 stage III patients (log-rank, *P* = 0.18; Figure 1D). There was no significant difference between *Smad4* LOH status according to demographics and most clinical features. There was, however, a borderline statistically significant association for tumor location (p = 0.05) (Table 1). LOH-positive tumors were more likely to be in the proximal (44.1%) and distal (36.4%) colon than in the rectum (19.5%, Table 1).

### Analysis of *Smad4* mutations

**Figure 2:**
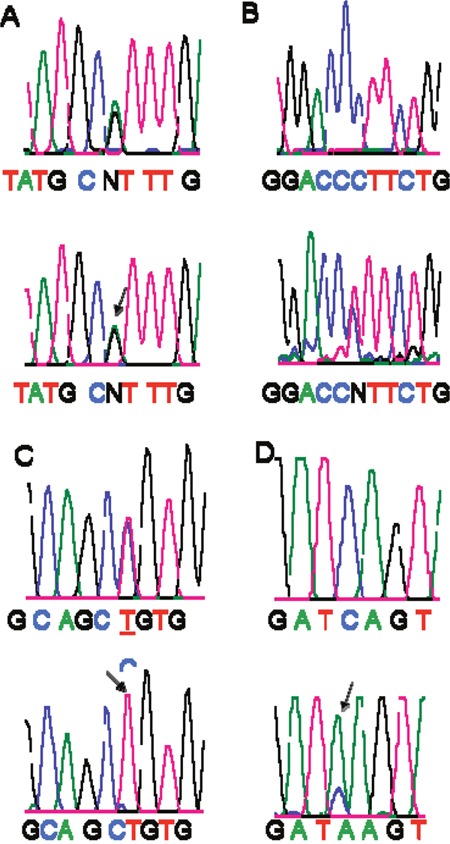
Detection of *Smad4* gene mutations A silent mutation, GCG (Ala) to GCA (Ala) at codon118 **Panel-A**. A missense mutation, CCT (Pro) to CTT (Leu) at codon 356 **Panel-B**. A silent mutation, GCC (Ala) to GCT (Ala) at codon 464 **Panel-C**. A missense mutation, TCA (Ser) to TAA (stop) at codon 474 **Panel-D**. *Arrows* show the mutations. Tumor tissues are below, and corresponding normal tissues are above.

A set of primers that covered the entire *Smad4* coding region (exon 1 through 11; codons 1 through 552) was used to screen for *Smad4* mutations by direct sequencing after PCR amplification. Among 53 samples, only 4 *Smad4* mutations were found (stage I, 5; II, 18; III, 21; IV, 9) (4/53, 7.5%). Genomic DNA was extracted from the corresponding FFPE tissues that exhibited *Smad4* mutations that had been confirmed by exon (2, 8 10)-specific primers and direct DNA sequencing. Of these four mutations, there were two missense mutations (codon 356 and 474) leading to changes in the amino acid residues and two silent mutations (codon 118 and codon 464) (Figure 2). The four mutations were all in DNA from Caucasian patients; three of these were male. The 53 cases (21 LOH positive, 28 LOH negative, and 4 not informative) were analyzed for their mutational status; there were no *Smad4* mutations in LOH-positive cases (2 were LOH-negative, 2 were not informative). Due to the small number of mutations, associations according to tumor stage could not be determined. These findings indicate that, in CRCs, *Smad4* may not be a target for mutational inactivation.

### Bivariate associations

The bivariate associations of SMAD4 characteristics, demographic variables, and tumor-related measures are shown in Table 2. For all study participants, LOH of *Smad4* was associated with increased hazard of death, however, this association is statistically not significant (HR = 1.15; 95% CI, 0.78-1.71). Low nuclear expression of SMAD4 protein was also associated with an increased hazard (HR = 1.44; 95% CI, 0.97-2.12) of marginal statistical significance. For all stages, cytoplasmic expression of SMAD4 protein was not associated with mortality.

**Table 2:** Bivariate associations with colon cancer-specific death

Study characteristics	Unadjusted HR (95% CI)
***18q21* deletion**	
**no LOH**	Ref
**LOH**	1.15 (0.78, 1.71)
**Cytoplasmic expression**	
**Low**	Ref
**High**	0.78 (0.52, 1.19)
**Nuclear expression**	
**High**	Ref
**Low**	1.44 (0.97, 2.12)
**Age**	
**< 65**	Ref
**≥ 65**	0.91 (0.62, 1.35)
**Sex**	
**Male**	Ref
**Female**	1.57 (1.06, 2.32)
**Race**	
**Caucasian**	Ref
**African American**	1.21 (0.82, 1.79)
**Tumor stage**	
**Stage I**	Ref
**Stage II**	1.71 (0.73, 3.96)
**Stage III**	3.88 (1.73, 8.72)
**Stage IV**	10.95 (4.80, 24.99)
**Tumor size**	
**≤ 65**	Ref
**> 65**	0.93 (0.58, 1.49)
**Tumor grade**	
**Low**	Ref
**High**	1.71 (1.14, 2.58)
**Tumor location**	
**Rectum**	Ref
**Distal colon**	1.84 (1.02, 3.32)
**Proximal colon**	1.70 (0.97, 2.99)

HR, hazard ratio.

### Multivariate results

The associations for *Smad4* LOH and immunophenotypic expression of this protein with death due to CRC are shown in Table 3. This table depicts the overall association of LOH and phenotypic expression of the protein as well as stage-specific associations after adjustment for *Smad4* status, tumor stage, tumor grade, and location. Overall, LOH was not associated with CRC-specific death (HR = 1.45; 95% CI, 0.91-2.31). However, it was associated with a 3.3-fold increased hazard of death for stage II patients that was of borderline statistical significance (HR = 3.30; 95% CI, 0.93-11.69). Although there was an association for stage I patients, this relationship failed to reach statistical significance due to only seven events (deaths due to CRC) among these patients. Cytoplasmic protein expression was not associated with mortality for all stages combined. However, among stage II patients, a high cytoplasmic protein level was associated with a 4.7-fold (HR = 4.71; 95% CI, 0.98-22.65) increased hazard of cancer-specific death that was borderline statistically significant. Overall, low nuclear protein expression of SMAD4 was associated with a 1.7-fold (HR = 1.70; 95% CI, 0.96-3.00) increased hazard of death that was of marginal statistical significance. The lack of nuclear protein expression was primarily confined to stage III patients, who showed a 2.7-fold difference (HR = 2.67; 95% CI, 0.98-7.32) that was also borderline statistically significant (Table 3).

**Table 3:** Association of *Smad4* allelic status and phenotypic expression with overall mortality and by tumor stage

Variable	Overall HR^1^ (95% CI)	Stage I HR^1^ (95% CI)	Stage II HR^1^ (95% CI)	Stage III HR^1^ (95% CI)	Stage IV HR^1^ (95% CI)
***18q21* deletion**					
**no LOH**	Ref	ref	ref	ref	ref
**LOH**	1.45 (0.91, 2.31)	5.66 (0.59, 54.52)	3.30 (0.93, 11.69)	1.20 (0.58, 2.51)	1.84 (0.70, 4.85)
**Nuclear expression**					
**High**	Ref	ref	ref	ref	ref
**Low**	1.70 (0.96, 3.00)	0.28 (0.03, 2.34)	2.09 (0.72, 6.10)	2.67 (0.98, 7.32)	0.44 (0.11, 1.79)
**Cytoplasmic expression**					
**Low**	Ref	ref	ref	ref	ref
**High**	1.39 (0.76, 2.56)	1.34 (0.08, 21.48)	4.71 (0.98, 22.65)	1.22 (0.44, 3.39)	0.65 (0.17, 2.44)
**Tumor grade**					
**Low**	Ref	ref	ref	ref	ref
**High**	1.80 (1.15, 2.83)	0.48 (0.03, 6.68)	1.56 (0.58, 4.20)	1.28 (0.62, 2.65)	5.80 (2.25, 14.94)
**Tumor location**					
**Rectum and distal colon**	Ref	ref	ref	ref	ref
**Proximal colon**	1.72 (1.11, 2.67)	0.91 (0.11, 7.81)	1.50 (0.59, 3.79)	1.39 (0.68, 2.84)	7.56 (2.18, 26.29)
**Tumor stage**					
**Stage I**	Ref				
**Stage II**	1.61 (0.67, 3.84)				
**Stage III**	3.20 (1.39, 7.36)				
**Stage IV**	11.92 (4.99, 28.48)				

^1^Adjusted for the variables listed.

HR, hazard ratio;' CI, confidence interval.

## DISCUSSION

First identified at the *18q21* locus in 1996 [3], *Smad4* and has attracted interest as a candidate tumor suppressor gene for *18q21* allelic imbalance. Various studies have demonstrated associations between *Smad4* mutations, SMAD4 protein expression, and *18q21* allelic imbalance at the *Smad4* locus [10, 11, 32]. The results, however, have not been consistent. The prevalence of *Smad4* mutations is about 50% [33, 34] in pancreatic carcinomas, but, in other tumor types, these mutations are less frequent [16, 35–38].

In CRCs, the frequency of loss of SMAD4 expression ranges from 9 to 67% [27, 39, 40]. Loss of SMAD4 expression in CRCs is associated with advanced-stage disease, presence of lymph node metastasis, and poor prognosis [15, 17, 24, 25, 27, 31]. Xie et al [25]. reported that loss of or reduced SMAD4 expression was associated with significantly shorter overall survival, but Kouvidou et al. [28] failed to find this relationship. The latter investigators demonstrated a significant correlation only between SMAD4 expression and tumor grade. Seshimo et al. [16] found that loss of SMAD4 expression was more frequent in poorly differentiated adenocarcinomas than in well- and moderately-differentiated carcinomas. In contrast, Bacman et al. [41] observed that lack of SMAD4 nuclear expression was not correlated with tumor grade or clinical outcome. SMAD4 expression is retained in high-grade CRCs, suggesting that loss of SMAD4 is a late event in carcinogenesis [28]. However, other investigators find no influence of reduced SMAD4 expression on prognosis [41]. There is a poor prognosis for CRCs with SMAD4, and high SMAD4 expression predicts a better prognosis for CRC patients with curative surgery [42]. Also, loss of SMAD4 expression is an independent prognostic factor, because it is associated with recurrence-free survival and overall survival in CRC patients [43]. In the current study, SMAD4 protein immunophenotic expression levels in the nuclear and cytoplasmic compartments of malignant cells were analyzed by IHC for 209 CRC patients with various stages of disease. Of these patients, 37% had low SMAD4 expression in the nuclei, whereas 26% had low SMAD4 expression in the cytoplasm. These proportions are in agreement with those of other studies [11, 28, 35, 39, 41, 44–49], indicating that *Smad4* is a tumor suppressor gene and that its protein expression, especially in nuclei, is lost in most cases of CRC progression. Kaplan-Meier and multivariate analyses found poorer overall survival for stage III patients with low nuclear SMAD4 protein. In contrast, among stage II patients, high cytoplasmic expression of the protein was associated with worse survival. Further, a borderline significant shorter survival was found for those with low SMAD4 protein levels in the nuclei with *Smad4* LOH positive in stage III CRC patients, indicating that *Smad4* is a tumor suppressor gene for these *18q21* deletions at the *Smad4* locus. SMAD4 levels decrease progressively from stage I through stage IV CRC, and the incidence of SMAD4 loss is higher in advanced stages of CRC [50, 51]. Our results demonstrate that low nuclear SMAD4 tumor protein in stage III and high cytoplasmic protein levels in stage II tumors are markers of poor prognosis for CRC patients. These findings can be considered in relation to reports that loss of expression of SMAD4 is more frequent for stage III than for stage II CRCs, that stage III CRCs that are microsatellite stable and exhibit loss of SMAD4 have a worse prognosis, and that time to recurrence after curative therapy is shorter for patients with stage III CRCs with SMAD4 loss [43, 50]. Together, these results can be useful in stratifying and predicting outcomes for patients with stage II or stage III CRC.

SMAD4 acts as a central mediator in the TGF-ß superfamily signaling pathway. The receptor-regulated SMADS, i.e., phosphorylated SMAD2 and SMAD3, are mediated by SMAD4 to translocate into the nucleus, where they are involved in the regulation of expression of genes that are necessary for cell proliferation, differentiation, and apoptosis [52–54]. In the absence of TGF-ß signaling, SMAD4 shuttles between the cytoplasm and nucleus, suggesting that nuclear SMAD4 has a function in unstimulated cells [55]. In addition to the role of mediating and carrying SMAD2 and SMAD3 into the nucleus, SMAD4 may also cooperate with these factors in regulation of transcription within the nucleus [56]. SMAD4 is directly or indirectly involved in regulation of genes involved in cellular invasion, e.g., those for E-cadherin, P-cadherin [57], and UK Pan-1 [58]. In addition, SMAD4 may induce the expression of cyclin-dependent kinase inhibitors, including p21, p27, and p15 [59, 60]. This induction regulates the proliferation and apoptosis of cells [61]. Abnormally low SMAD4 IHC levels in the nucleus could indicate the interruption of shuttling and cause a deficiency in mediating nuclear translocation of other SMADS or in regulating gene expression in a more direct way. These functions of SMAD4 in nuclei may explain the association between the low levels of SMAD4 in the nuclei and poorer clinical outcomes for CRC patients, especially for those with stage III disease.

Deletions in the arm of chromosome 18q are among the most common genetic abnormalities found in CRCs [4, 62]. The relationship between mortality and loss of chromosome genetic material, however, is inconsistent. Some reports show that allelic loss of chromosome *18q21* has a negative impact on prognosis and survival; [5, 7, 29, 31, 63–66] other studies find no prognostic value [5, 8, 9, 43, 67, 68].

In the current study of 193 informative cases, 118 cases (61%) showed LOH of *18q21* at the *Smad4* locus. This ratio is in agreement with a previous report [46]. In all 209 CRC cases (all stages included), there was no significant difference in survival between the cases with and without LOH of *Smad4*. However, an association between *Smad4* LOH and mortality was demonstrated for stage II CRC patients in both bivariate and multivariate analyses. This finding is in agreement with previous reports [11, 51] suggesting that *Smad4* LOH in stage II CRCs is a prognostic marker for cancer recurrence. For these high-risk stage II patients, the benefit of adjuvant chemotherapy following surgery should be assessed.

In cancer cells, allelic deletion of *Smad4* is a mechanism attributed to the loss of TGF-β tumor-suppressive activities. As CRC progresses into later stages, TGF-ß switches from functioning as a tumor suppressor to a tumor promoter, and SMAD4 loss may be involved in this shift [69–71]. In CRCs and pancreatic cancers, allelic losses have a prevalence of approximately 40% to 55% [72, 73]. In normal epithelial cells and early-stage neoplasms, SMAD4 functions as a tumor suppressor [74]. When an allele is partially or completely lost, other intact allele generally compensate for the loss. In some cases, however, the mutant allele does not function normally and either directly inhibits the activity of the wild-type protein or inhibits the activity of another protein that is required for the normal function of the wild-type protein (a dominant-negative mutation) [75]. In other cases, after one functional allele is altered, the other normal allele cannot produce enough protein, a state (haploinsufficiency) that leads to disease [75].

As determined in the current study, only stage II patients with LOH of *Smad4* demonstrated shorter survival. In addition, high cytoplasmic protein was associated with an increased risk of death for stage II patients, and, for stage III patients, low nuclear SMAD4 IHC expression was associated with an increased risk for cancer-specific mortality. Allelic imbalance of *18q21* at the *Smad4* locus may be only a factor for early CRC progression and not a necessary cause for metastatic progression.

The present effort found only 4 somatic mutations in 53 tumors, a finding in agreement with the low number of mutations previously reported [10, 11, 16, 17, 45, 46]. Therefore, *Smad4* mutational alternations appear to be involved only to a minor extent in CRC development.

Although adjuvant chemotherapy is considered efficacious for Stage III tumors, it is controversial for Stage II cancer patients [76]. Patients with stage III tumors with retained 18q who underwent adjuvant chemotherapy had a five-year, disease-free survival (DFS) advantage compared to those with LOH at *18q* [77]. In a retrospective analysis of patients treated in two adjuvant trials (ECOG 2284 and INT 0035) for high-risk Stage II and Stage III colon cancer, patients with tumors containing 18q LOH were significantly associated with worse DFS [77]. However, other retrospective studies of adjuvant colon trials, CALGB 9581 and CALGB 89803, did not demonstrate this difference in survival [78]. Currently, a prospective study, ECOG 5202 (NCT00217737), is assessing the clinical utility of *18q* LOH in recurrent stage II CRC. (https://clinicaltrials.gov/ct2/show/NCT00217737)

For CRCs, SMAD4 molecular alterations were predictive of failure to respond to fluoropyrimidine-based treatment [79] and of development of liver metastases [80]. Loss of SMAD4 in CRC tumors appears to induce resistance to 5FU-based therapy through regulation of a downstream apoptotic pathway and upregulation of PI3K/Akt and VEGF [14, 79, 81]. These findings were validated in other retrospective studies [81]. Thus, tumors with SMAD4 LOH may benefit from adjuvant treatment with non-5FU based regimens. Since assessing this molecular alteration to monitor the treatment in real-time may be challenging, the IHC findings of the present study will be useful in assessing Smad4 LOH as candidate predictive biomarker for therapeutic application.

In summary, LOH of *Smad4* and high cytoplasmic protein in Stage II and low nuclear SMAD4 protein localization in Stage III CRCs are useful predictors of shortened patient survival.

## MATERIALS AND METHODS

### Patients and samples

The Institutional Review Board of the University of Alabama at Birmingham (UAB) approved this study. All samples were supplied by the Tissue Procurement Facility of the UAB Comprehensive Cancer Center. Formalin-fixed, paraffin-embedded (FFPE) archival tissue blocks were obtained for 209 CRC patients (Stage I, 34; Stage II, 70; Stage III, 64; Stage IV, 41) who had undergone surgical resection for a first primary sporadic CRC at the UAB Hospital from 1981 through 2002. None of these cases are Lynch syndrome patients and the microsatellite status (MSI) of these cases was not evaluated. Of the 209 cases, 53 had frozen tissues available (both CRCs and their corresponding normal control tissues).

The medical records of these patients were reviewed by two investigators (CKS & RH), and the surgical pathology reports were reviewed by two GI pathologists (CKS & NCJ). During the initial selection process, the following patients were excluded from the study population: those who died within a week of their surgery, those with inflammatory bowel disease, those with surgical-margin involvement or unspecified tumor location, those with multiple primary cancers within the colorectum or multiple malignancies, and those with a family or personal history of CRC. To control for treatment bias, those patients who underwent only surgery as a therapeutic intervention were included, and those who those who received any pre- or post-surgical chemotherapy were excluded.

Two GI pathologists (CKS & NCJ) independently reviewed slides (stained with hematoxylin and eosin) to determine the degree of histological differentiation of CRC tumors and, if there were discrepancies, reached a consensus. As in an earlier study [82], well and moderately differentiated CRCs were classified as “low” grade, and poorly differentiated tumors were classified as “high” grade pathological staging was performed according to the criteria of the American Joint Commission on Cancer (stages I, II, III, and IV) [83]. The International Classification of Diseases for Oncology (ICD-O) codes were used to specify anatomic locations of the tumors [84]. The anatomic sites were designated as proximal colon (cecum, ascending colon, and proximal 2/3 of the transverse colon), distal colon (distal 1/3 of the transverse colon, descending colon, and sigmoid colon), and rectum.

Patients were followed by the UAB Tumor Registry until their death or until the date of the last documented contact within the study time frame. The Tumor Registry ascertains outcome (mortality) information directly from patients (or living relatives) and from the physicians of the patients through telephone and mail contacts. This information is validated against State Death Lists. The Tumor Registry updates follow-up information every six months. Follow-up of the cohort ended in November 2008.

### SMAD 4 immunohistochemical (IHC) staining

Tissue sections (5-μm) were cut from paraffin blocks 1-3 days prior to immunostaining to avoid potential problems in antigen recognition due to storage degradation of cut tissue sections on glass slides [85]. Immunostaining was performed as described in our earlier studies of antigen expression in various tissues [29, 30]. In brief, the sections were deparaffinized in xylene and rehydrated in graded alcohol. For antigen retrieval, the tissue sections in citrate buffer were boiled in a microwave oven, and the sections were treated with 3% H_2_O_2_ for 5 minutes to block endogenous peroxidase activity. After 1 hour of blocking with 3% goat serum, slides were incubated for 1 hour with 1:100 dilution of an anti-human monoclonal SMAD4 antibody (clone B-8; sc-7966; Santa Cruz Biotechnology Inc., Santa Cruz, CA, USA). The specificity of this antibody has been established [32–33]. Sections on which the primary antibody was not applied were utilized as negative controls. Secondary detection was accomplished with a multi-species detection system (Signet Lab Inc., Dedham, MA). The sections were exposed to biotinylated multispecies antibodies, including anti-mouse antibodies, for 20 minutes and then incubated with peroxidase-labeled streptavidin for 20 minutes. A diaminobenzidine tetrachloride super-sensitive substrate kit (BioGenex, San Ramon, CA) was used to visualize the antibody-antigen complexes. Each section was then counterstained with hematoxylin, dehydrated with graded alcohols, and soaked in xylene before application of coverslips.

**Figure 3:**
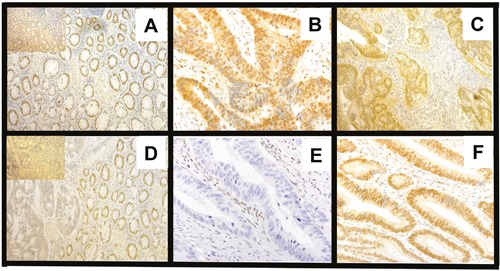
SMAD4 immunostaining patterns in CRCs based on *Smad4* LOH status **A**. Normal strong positive (200 μm); inset, control (without primary antibody) **B & C**. CRCs with cytoplasm and nuclei both high (600, 400 μm) **D**. Normal positive, tumor cytoplasm low and nuclei negative. Inset, control **E**. Tumor, cytoplasm and nuclei negative **F**. Tumor, with nuclei positive

The evaluation of staining of SMAD4 expression was as described in our earlier studies [29, 30]. To limit bias, evaluations were performed by two pathologists. In all cases, normal colon mucosa displayed positive staining, and both nuclear and cytoplasmic staining were evident. For tumor cells, the percentages of positive cells were evaluated separately for the nucleus and cytoplasm. In normal colonic epithelial tissues, strong IHC staining in both the nucleus and cytoplasm was evident. A semiquantitative immunostaining score (ISS) for SMAD4 was obtained by two pathologists who estimated the proportion of cells stained and the intensity of staining in the whole tissue section [86]. The intensity of immunostaining of individual cells was scored on a scale from 0 (no staining) to 4 (strongest intensity). Scores derived by the two investigators were combined to obtain average ISS values. The median staining value obtained for normal colon mucosa was 40%. An ISS of ≥ 0.5 and ≥ 40% percent of positive cells was chosen as a cut-off value for determining high vs. low expression of SMAD4. Based on the extent of SMAD4 staining in control tissues, the cut-off value was ≥ 0.5 for both the nucleus and cytoplasm. By use of this cut-off value and the percentage of positive cells (40%), the 209 CRC cases were separated into two groups: low protein expression of SMAD4 (< 0.5, < 40%) and high protein expression of SMAD4 (≥ 0.5, ≥ 40%). Representative pictures of SMAD4 immunostaining patterns are shown in Figure 3.

### LOH analysis at the *Smad4* locus

Three polymorphic microsatellite markers (D18S363, D18S474, and D18S46), which are within 0.3 Mb of the *Smad4* gene in the *18q21.1* region, were used to analyze the LOH status. A description of these markers is in Table 4. Samples of genomic DNA (~100 ng) extracted from CRCs and matching normal tissues were used as templates in the polymerase chain reaction (PCR). The PCR reaction volume (25 μl) contained 10X PCR buffer, 1 mM of each dNTP, 15 mM of MgCl_2_, 10 pmoles of each marker, and 0.3 μl (1.5 units) of Platinum Tag Polymerase (Invitrogen Life Technologies, Carlsbad, CA). Amplification proceeded under the following conditions: 94°C for 5 minutes, 35 cycles of 94°C for 30 seconds, 55°C for 30 seconds, and 70°C for 1 minute. The final extension was 70°C for 7 minutes. The PCR product (2 μl) was mixed with 1 μl of GeneScan-500 ROX standard (Applied Biosystems, Foster City, CA) in 12 μl of Hi-Di formamide (Applied Biosystems), denatured for 5 minutes at 88°C, and cooled on ice. The samples were subjected to capillary electrophoresis with an ABI PRISM™ 3100 Genetic Analyzer (Applied Biosystems). The results were analyzed with Genotyper 2.1 software (Applied Biosystems). Incomplete allelic losses were commonly observed because of contamination by normal cells or tumor heterogeneity. As previously described [87], one of the tumor alleles decreased by > 40% when the calculated ratio was >1.49 or < 0.5; thus, we counted this ratio value as an indication of LOH positivity. Homozygous cases were considered as non-informative for LOH.

$${\text{LOH Ratio}}^{=}\frac{\text{Tumor allele }1/\text{tumor allele }2}{\text{Normal allele }1/\text{normal allele}2}$$

Table 4Three markers of LOH at the *Smad4* locus

**Table:** 

D18S46	F: HEX GAA TAG CAG GAC CTA TCA AAG AGC
	R: CAG ATT AAG TGA AAA CAG CAT ATG TG
D18S363	F: 6FAM TTG GGA ACT GCT CTA CAT TC
	R: GCT TCA TTC TCT CAC TGG AT
D18S474	F: NED TGG GGT GTT TAC CAG CAT C
	R: TGG CTT TCA ATG TCA GAA GG

**Table:** Sequences of primers for direct sequencing of *Smad4*

*Smad4P1F*	ATGGACAATATGTCTATTACGAAT
*Smad4P2F*	GACAGCCATCGTTGTCCA
*Smad4P3F*	AGGTAGGAGAGACATTTA
*Smad4P4F*	TGCACCTGGAGATGCTGT
*Smad4P1R*	ACCTCAGTCTAAAGGTTGTGGGTC
*Smad4P2R*	CACCTTTGCCTATGTGCA
*Smad4P3R*	CAACTCGTTCGTAGTGAT

**Table:** Sequence of RT-PCR for expression of *Smad4* mRNA

*Smad4*	F: GTGTGAATCCATATCACTACGAACG
	R: CATACTTGATGGAGCATTACTCTGC
β-Actin	F: TAAGTAGGCGCACAGTAGGTCTGA
	R: AAGTGCAAAGAACACGGCTAAG

### Mutation analysis of *Smad4*

### RNA extraction, RT-PCR, and PCR amplification

Easy Mini RNA isolation kits (Qiagen, Valencia, CA) were used to isolate RNA from frozen tissues. The cDNAs were prepared from purified RNA (~100 ng/μl) with 200 units/μl of superscript III (Invitrogen Life Technologies), 4 μl 5x RT buffer, 1 μl of 10 mM dNTP mixture, 1 μl of 50 μm oligo dT, 2 μl of 0.1M DTT, 4 μl of 25 mM MgCl_2_, and 40 units/μl of RNase OUT. The reverse transcription reaction was performed by incubating samples at 50°C for 50 minutes and then heating at 70°C for 15 minutes to inactivate superscript III. The RNA template from the cDNA-RNA hybrid molecule was removed by digesting with RNase H at 37°C for 20 minutes. The final volume of the cDNA reaction mixture was 25 μl. cDNA (6 μl) was used as a template to amplify the *Smad4* gene by PCR. The forward primer sequence was 5′-ATGGACAATATGTCTATTACGAAT-3′, and the reverse primer sequence was 5′-ACCTCAGTCTAAAGGTTGTGGGTC-3′.

### Mutational analysis by direct DNA sequencing

The resulting PCR products were sequenced by use of an ABI 3100 sequence detector with a set of primers covering the entire *Smad4* coding region (exon 1 through 11; codons 1 through 552). The set included four forward primers and three reverse primers (Table 4). To detect gene mutations in *Smad4*, sequencing data were analyzed with DNA Star software.

### Genomic DNA extraction from paraffin blocks and PCR with specific exon primers

To detect mutations, paraffin sections of tissue blocks of CRCs and their matched normal samples were examined under a microscope, and genomic DNA samples were extracted. This was followed by PCR amplification using exon-specific primers and direct DNA sequencing. The PCR forward primer sequence was 5′ –AGGCATTGGTTTTTAATGTATG-3′, and the reverse primer sequence was 5′-CTGCTCAAAGAAACTTAATCAAC-3′ (exon 10).

### Statistical analyses

Characteristics of the study population were compared according to their *Smad4* LOH status and *Smad4* protein expression in the nucleus and cytoplasm. Chi-square statistics were used to determine the statistical significance of the observed versus expected distributions of demographic and tumor variables. Kaplan-Meier overall survival curves were plotted for LOH status and for protein expression by use of PRISM software (GraphPad Software, Inc., La Jolla, CA). Patients were stratified into phase II and phase III groups, and log-rank tests were used to determine if survival differed by levels of nuclear protein expression, cytoplasmic expression, and LOH status.

The Cox proportional hazards model was used to obtain hazard ratios (HRs) with 95% confidence intervals (CIs) for the bivariate association of *Smad4* LOH, phenotypic protein expression of SMAD4, and other covariates with death due to CRC. Cox multivariate models were constructed to obtain adjusted HRs for the association of *Smad4* (LOH and phenotypic expression) with cancer-specific mortality. For selection of variables, in addition to tumor stage, those variables associated with mortality at *p* < 0.20 in the bivariate analysis were considered potential confounders for the association of SMAD4 with mortality and were included in the initial multivariate model. To obtain the final model, the least significant variable was removed in a step-wise manner. Once the final model was obtained, the statistical significance of all two-way interactions between LOH status, nuclear expression, and tumor stage was assessed. The proportional hazards assumption was evaluated and met for *Smad4* LOH and protein expression of SMAD4. Patients were again stratified by stage into four subgroups, and similar multivariable analyses were accomplished for each group.
